# Patient‐specific visco‐hyperelastic mechanical model for breast tumor localization in surgical planning

**DOI:** 10.1002/btm2.70044

**Published:** 2025-07-09

**Authors:** Felicia Alfano, Pedro Navas, Pablo Lamata, Karla Ferreres García, Juan E. Ortuño, Oscar Bueno Zamora, Santiago Lizarraga, Andrés Santos, Javier Pascau, José M. Goicolea, María J. Ledesma‐Carbayo

**Affiliations:** ^1^ Biomedical Image Technologies Universidad Politécnica de Madrid Madrid Spain; ^2^ CIBER de Bioingeniería, Biomateriales y Nanomedicina, Instituto de Salud Carlos III Madrid Spain; ^3^ Department of Continuum Mechanics and Theory of Structures Universidad Politécnica de Madrid Madrid Spain; ^4^ School of Biomedical Engineering and Imaging Sciences King's College London UK; ^5^ Instituto de Investigación Sanitaria Gregorio Marañón Universidad Carlos III de Madrid Madrid Spain

**Keywords:** breast cancer, preoperative localization, surgical planning, visco‐hyperelasticity

## Abstract

Breast‐conserving surgery is typically performed with the patient in a supine position, whereas preoperative diagnostic MRI breast images are obtained with the patient in a prone position. The change in patient positioning causes significant large deformations, requiring preoperative localization of the detected lesions. Developing an individual‐specific breast biomechanical model capable of simulating these deformations remains challenging yet highly desirable. This study presents a novel approach that combines finite element analysis with the optimization of mechanical properties of breast tissues, using only surface information to construct a personalized deformation model of the breast. A visco‐hyperelastic model is employed to characterize the stress–strain relationship of breast tissue. The proposed method has been tested on 15 cases of breast cancer and achieves a tumor localization error of 8.12 ± 4.15 mm. The results show that this approach provides an accurate and realistic estimation of large breast tissue deformations and yields smaller tumor localization errors compared to previously reported methods.

**Translational Impact Statement**

This study addresses a critical challenge in breast‐conserving surgery: the deformation of breast tissue due to differences in patient positioning during imaging (prone) and surgery (supine). By developing a personalized, visco‐hyperelastic biomechanical model that accurately estimates these deformations, we provide a practical tool for preoperative tumor localization. Tested on a diverse dataset encompassing various breast sizes, our approach achieves a significant reduction in tumor localization error compared to existing methods.

## 1 INTRODUCTION

Breast cancer is the most common cancer among women globally, with 2.3 million new cases diagnosed in 2022.^1^ Advancements in imaging and the implementation of screening programs have greatly improved the detection of clinically occult, non‐palpable breast lesions, for which breast‐conserving surgery (BCS) is the preferred treatment approach.^2^ BCS aims to remove tumors with adequate surgical margins while preserving as much healthy tissue as possible. Magnetic resonance imaging (MRI), which accurately defines tumor size and extent in a prone position, is essential in preoperative planning. However, the supine position required for surgery introduces significant breast deformation, making it difficult to directly apply preoperative images during surgery. For this reason, the surgical excision of a non‐palpable breast lesion requires some form of breast localization device. Wire‐guided localization (WGL) is the most commonly used method due to the high efficacy and low cost, but it has several limitations, including potential wire migration and patient discomfort.^3^, ^4^, ^5^ In recent years, several alternative localization techniques, such as radioactive seed localization, radio‐occult lesion localization (ROLL), and intraoperative ultrasound, have emerged, though these are not yet widely adopted due to cost and logistical constraints.^6^ The challenges in the current localization techniques have driven the exploration of alternative, non‐invasive techniques, such as medical imaging‐based approaches, to improve localization and surgical planning.^7^

Biomechanical breast models, mainly based on finite element (FE) methods, have been extensively studied for applications such as simulating breast behavior under compression during mammography, 2D–3D image registration, and surgical planning.^8^, ^9^, ^10^, ^11^, ^12^, ^13^, ^14^, ^15^, ^16^, ^17^, ^18^, ^19^, ^20^, ^21^ While FE models have shown promise in aligning preoperative images with the surgical situation, existing approaches often require additional imaging modalities, such as supine MRI or CT.^14^, ^15^, ^21^ Supine MRI, although providing valuable insights into breast tissue deformation, is a costly and time‐intensive procedure with no added diagnostic benefit. Moreover, non‐rigid deformation often occurs between the supine MRI position and the actual surgical positioning, necessitating further corrections during intraoperative planning.^22^ As a result, alternative, low‐cost, and practical methods are highly desirable. Other groups have proposed using intraoperative surface data acquired via optical scanners as a cost‐effective alternative to supine imaging. These surface scans can serve as boundary conditions for finite element models, enabling deformation of preoperative prone MRI volumes to match the surgical setup. Various groups have demonstrated the feasibility of this approach, employing external skin fiducials or anatomical landmarks to guide the deformation.^23^, ^24^ Other methods leverage preoperative CT in the supine position to guide surface‐based registration algorithms and FE‐based biomechanical models.^21^, ^25^

Breast tissue exhibits complex mechanical behavior, including non‐linear, anisotropic, and time‐dependent responses, making accurate modeling crucial for clinical applications.^26^, ^27^ Traditional FE models often rely on linear elastic or nonlinear constitutive laws to simulate breast deformation.^13^, ^21^, ^28^, ^29^ However, linear elastic models tend to perform poorly when large deformations are involved, which is a key characteristic of breast tissue deformation.^30^ As such, hyperelastic models, which can more accurately describe large deformations, have become the preferred choice in recent literature.^15^, ^16^, ^17^, ^21^ While hyperelastic models effectively describe large deformations, they lack the capability to account for time‐dependent responses, which are critical for modeling soft tissue behavior. Viscoelastic models incorporate time‐dependent material behavior, while hyperelasticity captures non‐linear deformation mechanics. Combining these principles into visco‐hyperelastic models provides a robust framework to simulate the mechanical response of soft tissues.^31^, ^32^ When combined with hyperelastic formulations, the resulting visco‐hyperelastic models capture both the non‐linear deformation mechanics and time‐dependent material responses, enabling more physiologically accurate predictions of soft tissue behavior. Taylor et al. have extensively demonstrated the importance of such combined approaches in biomechanical modeling, underscoring their potential to advance the precision of clinical applications.^31^ Despite their advantages, visco‐hyperelastic models remain underutilized in addressing prone‐to‐supine deformation.

This study introduces a visco‐hyperelastic FE model to estimate breast deformation and localize tumors using preoperative prone MRI and intraoperative surface scans. By leveraging optical surface scanning, the surgical surface can be acquired seamlessly in the operating room, requiring no significant alterations to the preoperative protocol. Our primary contributions are as follows:

- *A visco‐hyperelastic model*: Combining non‐linear and time‐dependent mechanical behavior to predict prone‐to‐supine breast deformation and accurately localize tumors using surface data alone.
- *Comprehensive validation*: Evaluation across a diverse dataset of breast anatomies, ensuring robustness and generalizability.
- *Open‐access resources:* Including prone and supine breast meshes, fostering reproducibility and further advancements in breast deformation modeling.

## 2 METHODS

This work proposes a novel method for performing volume‐to‐surface registration for tumor localization in breast surgery planning, using the preoperative volume obtained from a prone T2 Spectral Attenuated Inversion Recovery (SPAIR) MRI and the intraoperative surface, which could ideally be acquired with an optical scanner. In this study, the intraoperative surface was obtained from CT data in the supine position, as the CT also allows us to evaluate the estimated position of the tumor with respect to the actual position in the intraoperative pose.

The breast is modeled using a visco hyperelastic material to accurately capture the significant breast deformation from the prone‐to‐supine position and localize the tumor in the surgical position.

**FIGURE 1:**
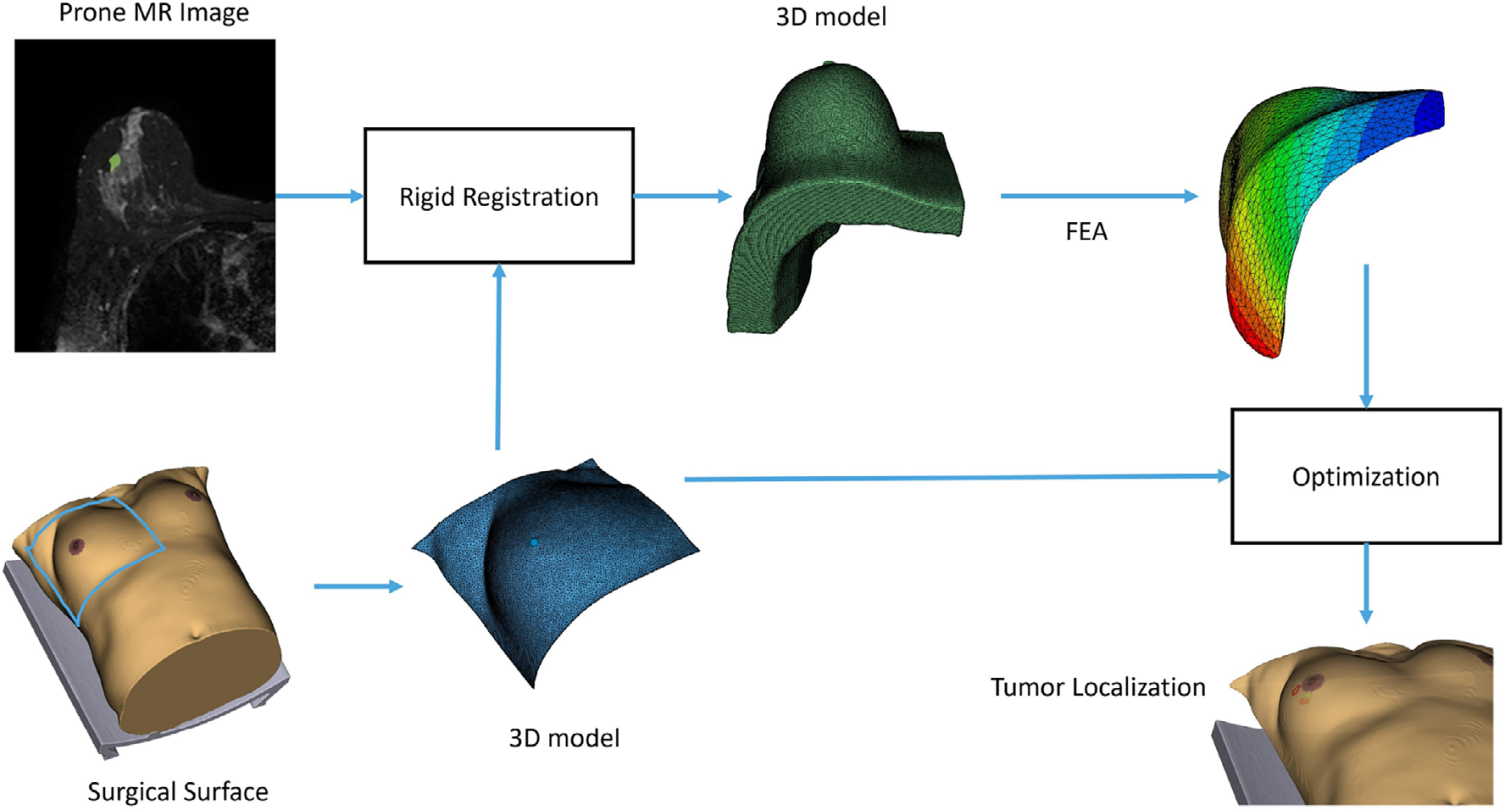
Overview of the proposed workflow, including rigid alignment between preoperative MRI and intraoperative surface data, finite element modeling, and patient‐specific material property optimization for prone‐to‐supine deformation simulation and tumor localization.

The methodology is structured into three main steps: (1) initial rigid alignment of the preoperative volume with the intraoperative surface, (2) finite element analysis (FEA), and (3) patient‐specific material property optimization. An overview of the workflow is presented in Figure 1.

### 2.1 Data description and preprocessing

The data for this study consisted of 15 retrospective cases of breast cancer selected from our cohort from the Hospital General Universitario Gregorio Marañón in Madrid, obtained with ethical committee approval. Each case included preoperative T2 SPAIR MRI and Subtraction MRI scans in the prone position, acquired several weeks before breast conserving surgery, and a CT image in the supine position, used to obtain the intraoperative surface and to locate the tumor for evaluation purposes. While the method is intended to work with actual intraoperative surface data (potentially from an optical scanner), we used CT data for this study to obtain the intraoperative surface as well as the tumor position.

The initial rigid alignment between the prone surface from the MRI and the supine surface was performed using the intermammary space as a reference, based on the assumption that this region remains relatively unchanged as breast movement occurs primarily in the posterior–lateral direction. A point‐based rigid registration algorithm was employed for this purpose.

The intermammary surface was automatically identified on the prone surface as the flat region between the breasts. On the supine surface, however, this area is more difficult to delineate. To address this, we measured the geodesic distance from the nipples to the boundary of the intermammary region on the prone surface and applied the same distance to identify the corresponding boundary on the supine surface.

To further refine alignment in the cranial‐caudal direction, we assumed that the nipples lie approximately at the same level in both positions. The quality of the initial alignment was visually assessed by a radiologist, ensuring that the surfaces were correctly aligned before proceeding to further steps.

Tumor positions in the prone MRI and supine CT were manually annotated with the supervision of an expert radiologist. The position of the tumor derived from the CT, and transformed by the rigid registration, was only used as the reference intraoperative tumor position for evaluation purposes.

Table 1 summarizes patient and tumor characteristics, including breast volume (BV), tumor diameter, and prone‐to‐supine tumor displacement.

**TABLE 1:** Patient and tumor characteristics.

Characteristic	Range	Mean
Age (years)	46–82	65
Breast volume (cm^3^)	718.1–3296.9	1738.6
Tumor diameter (mm)	4.5–33.6	15.0
Prone‐to‐supine tumor displacement (mm)	30.7–98.7	60.2

*Note*: This table presents key characteristics of the study population, including the age of the patients, breast volume, tumor diameter, and prone‐to‐supine tumor displacement.

For a more comprehensive evaluation, the 15 cases were stratified into three breast volume groups: small (BV ≤ 1600.0 cm^3^), medium (1600.0 < BV < 2000.0 cm^3^), and large (BV ≥ 2000.0 cm^3^). Each group consisted of five cases, ensuring a diverse representation of breast sizes to improve the reliability and generalizability of the proposed method. This stratification allows for better analysis of how breast size influences the accuracy and effectiveness of tumor localization during surgery. Larger breasts, in particular, can present more challenges in tumor localization due to increased deformation, highlighting the importance of evaluating the method across a range of breast sizes.

### 2.2 Finite element‐based breast biomechanical modeling

**FIGURE 2:**
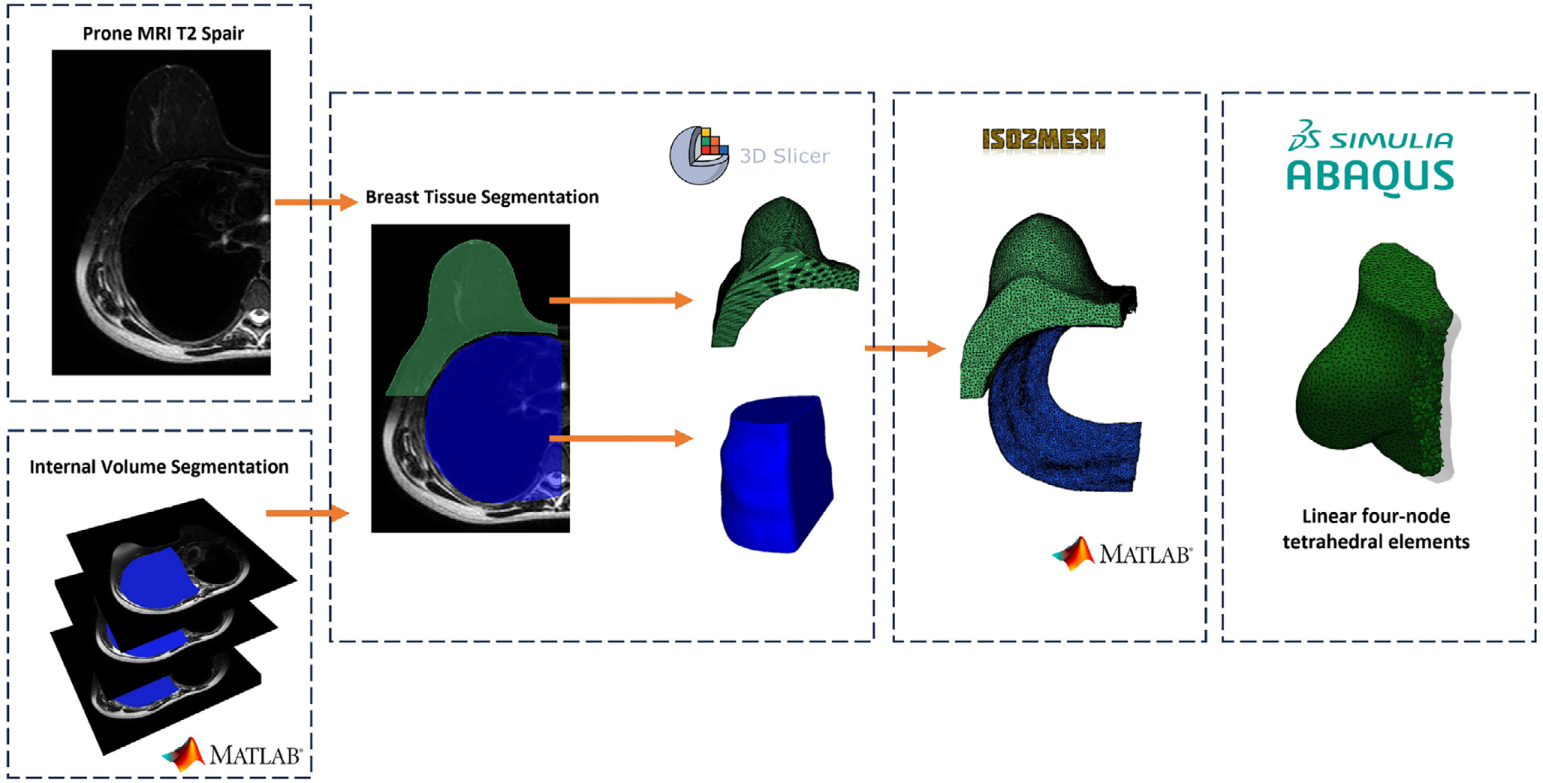
Workflow for generating tetrahedral meshes from preoperative MRI.

To obtain the patient‐specific breast model, the preoperative prone T2 SPAIR MRI images were segmented using 3D Slicer.^33^ For breast tissue segmentation, we initially applied thresholding followed by smoothing filters to enhance breast tissue delineation. However, the internal margin of the breast and its interface with the chest wall were indistinct in the T2‐weighted MRI images. To address this, we manually annotated the internal profile in representative slices and interpolated the results across the entire volume. The final segmentation of the breast tissue was obtained by subtracting the generated internal volume, resulting in a more accurate delineation of the breast at the interface with the chest wall. This step typically takes around 5 minutes per case. Since the skin is not clearly visible in MRI images, it was modeled as an extrusion of the breast's outer surface, with a thickness of 1.5 mm.^17^ Segmented volumes were exported as surface meshes and refined using the *iso2mesh* library.^34^ These surfaces were meshed into hybrid four‐node linear tetrahedral elements using ABAQUS (Version 6.22, Dassault Systèmes, 2022).^35^ The average number of tetrahedral elements assigned to the merged skin and breast tissue was 47,935, with an average edge length of approximately 6 mm. The complete workflow, from image segmentation to tetrahedral mesh generation, is illustrated in Figure 2.

### 2.3 Mechanical properties of breast tissue

Various modeling approaches have been used to represent the breast, ranging from simple models to more sophisticated ones that account for the different tissue types comprising the breast. The most basic approaches treat the breast as a homogeneous tissue,^13^, ^36^, ^37^ while more complex models consider it as heterogeneous, consisting of distinct tissue types. For example, the model proposed by Mïra et al.^38^ includes adipose tissue, glandular tissue, skin, muscle, ligaments, and fascia.

However, experimental work by Ruiter et al.^8^ has shown that different tissue models may not significantly impact the results, suggesting that simplifying assumptions can still provide satisfactory outcomes. Recently, Ringel et al.^39^ also reported that accounting for the heterogeneity of adipose and glandular tissue alone did not lead to substantially accuracy improvements.

In our work, we have adopted a simplified model that represents the breast using only two primary tissue types: the interior of the breast, which includes adipose tissue, fibro‐glandular tissue, pectoral muscle, and Cooper's ligaments (collectively referred to as “breast tissue”), and the skin.

To accurately capture the behavior of the breast tissue and skin under deformation, we employed a visco‐hyperelastic material model. This formulation is particularly well‐suited to model biological tissues, which typically exhibit both elastic (instantaneous) and viscous (time‐dependent) responses.^31^, ^32^, ^40^, ^41^, ^42^

For the hyperelastic behavior of the breast tissue, we employed the neo‐Hookean model, a widely used approach to model the stress–strain relationship of soft biological tissues. The strain energy potential for this model is given by:

(1) $$\Psi =\frac{\mu_{0}}{2}\;\left(\bar{I_{1}}-3\right)+\frac{2}{K_{0}}{\left(J^{\mathrm{el}}-1\right)}^{2},$$

where $\bar{I_{1}}$ is the first invariant (trace) of the isochoric right Cauchy–Green deformation tensor and $J^{\mathrm{el}}$ is the determinant of the deformation gradient. $\mu_{0}$ is the initial shear modulus and $K_{0}$ is the initial bulk modulus that was defined such that $K_{0}$≫ $\mu_{0}$ to assume a quasi‐incompressible behavior in accordance with previous studies.^26^, ^29^, ^32^, ^38^, ^43^, ^44^, ^45^

To model the time‐dependent behavior of the tissues, we used a visco‐hyperelastic formulation. This approach accounts for the stress relaxation effects observed in biological tissues, which exhibit relaxation over time under constant strain. The stress relaxation function is defined as:

(2) $$\begin{array}{c} \hat{\Psi }\;\left(\Psi ,t\right)=\int_{0}^{t}G\left(t-s\right)\;\frac{\partial \Psi }{\partial s}\mathrm{ds}\; \end{array},$$

where *G*(*t*) is the stress relaxation function, and it can be approximated using a Prony series:

(3) $$\begin{array}{c} G\left(t\right)=G_{\infty }+\sum_{k=1}^{K}G_{k}\;\exp\left(-\frac{t}{\tau_{k}}\right)\; \end{array}$$

Here *G*_∞_ is the long‐term modulus, *G*_*k*_ represents the modulus of each Maxwell element, and *τ*_*k*_ is the corresponding relaxation time. The relaxation time *τ*_*k*_ for each Maxwell element is given by:

(4) $$\begin{array}{c} \tau_{k}=\frac{\eta_{k}}{G_{k}}\; \end{array},$$

where *η*_*k*_ is the dashpot viscosity and *G*_*k*_ is the spring stiffness. This model, based on a generalized Maxwell model, is suitable for simulating the time‐dependent behavior of tissues in a way that matches their observed mechanical properties.

The relaxation modulus can be written in a dimensionless form as *g*_*k*_ = *G*_*k*_/*G*_0_, where *G*_0_ represents the instantaneous shear modulus of the generalized Maxwell model at time *t* = 0. Thus, the relaxation function can be written as follows:

(5) $$\begin{array}{c} G\left(t\right)=G_{0}\left(\;1-\sum_{k=1}^{K}g_{k}\;\left(1-\exp\left(-\frac{t}{\tau_{k}}\right)\right)\right)\; \end{array}.$$

The skin was also modeled as visco‐hyperelastic. Its hyperelastic component follows the neo‐Hookean formulation, where the shear modulus is scaled relative to the breast tissue's shear modulus *μ*_*B*_ by a fixed scaling factor *μ*_*S*_ = *f* · *μ*_*B*_.^46^

### 2.4 Simulation setup and boundary conditions

Breast deformation is traditionally treated as a static or quasi‐static problem, analyzed using either static implicit methods^46^, ^47^, ^48^ or dynamic explicit FE methods.^31^, ^37^, ^49^, ^50^ Static implicit methods rely on iterative solutions to nonlinear equations, whereas dynamic explicit methods solve partial differential equations incrementally over small time steps, avoiding the need to assemble tangent stiffness matrices. Although primarily intended for transient dynamic problems, dynamic explicit methods are well‐suited for quasi‐static processes when inertial forces are negligible.

In this study, we employed the dynamic FE solver in ABAQUS/Explicit to analyze the mechanical deformation of breast tissue under gravity. This solver was chosen for its robust performance when handling highly nonlinear deformations, complex contact interactions, and nearly incompressible materials such as soft tissue. In our experience, it is as precise as implicit solvers such as ABAQUS/Standard, with fewer convergence issues.

The deformation from prone to supine position was simulated by inverting gravity, without accounting for pre‐stress. A gravitational load of 9.8 N/m^3^ was applied in the y‐direction, and the density of both skin and breast tissue was set to 1000 kg/m^3^. The skin and breast tissue were modeled as a single deformable body, with no allowance for sliding between these two structures.

Reproducing realistic breast deformation near the chest wall is essential. While some studies assume a rigid attachment of the breast to the chest muscles,^9^, ^21^, ^29^, ^36^ others model sliding behavior at the chest wall interface.^14^, ^15^, ^37^, ^51^, ^52^ In this work, we adopted the latter approach by defining a surface‐to‐surface contact with a small sliding formulation. The chest wall was treated as a rigid surface and fixed to prevent rigid body displacement.

**FIGURE 3:**
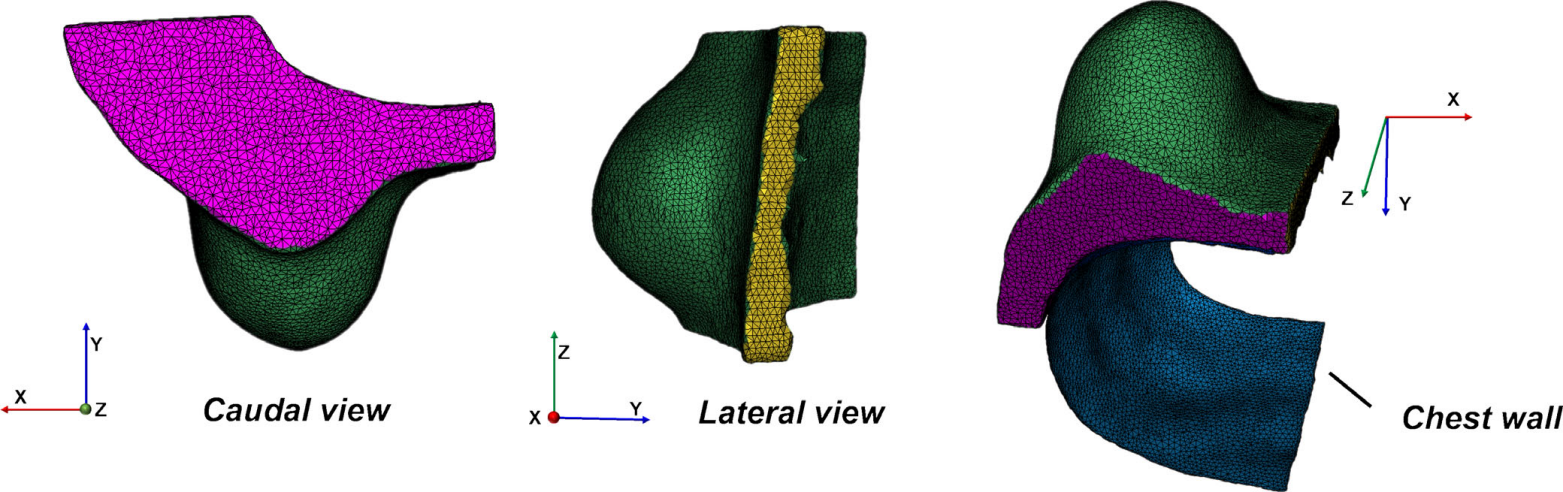
Finite element model of the breast and boundary conditions. Each color represents a specific boundary condition: The *Z*‐component of displacement is zero on the superior and inferior horizontal boundaries (magenta), while the *X*‐component of displacement is zero at the junction with the contralateral breast (yellow).Finite element model of the breast and boundary conditions. Each color represents a specific boundary condition: The *Z*‐component of displacement is zero on the superior and inferior horizontal boundaries (magenta), while the *X*‐component of displacement is zero at the junction with the contralateral breast (yellow).

The tumor‐affected breast was modeled in isolation, with symmetry boundary conditions applied at the junction with the contralateral breast. The *X*‐component of displacement was constrained to zero along the junction (yellow region in Figure 3), and the *Z*‐component of displacement was set to zero on the superior and inferior horizontal boundaries (magenta region in Figure 3).

Due to the lack of detailed anatomical boundary conditions in the postero‐lateral region, the model did not achieve static equilibrium. Instead, a stopping criterion was defined to determine the optimal deformation state: the time at which the simulated nipple position was closest to the actual nipple position. Across the 15 cases studied, the average simulation time was 0.161 ± 0.025 s, with a maximum simulation time of 0.3 s.

### 2.5 Patient‐specific optimization procedure

In this study, we employed a visco‐hyperelastic constitutive model to represent the behavior of the breast.

To the best of our knowledge, no published studies have comprehensively characterized the breast mechanical behavior using a visco‐hyperelastic model. Consequently, we lacked reference data regarding the viscoelastic properties of the breast. In contrast, the hyperelastic properties have been extensively studied, with reported shear modulus values ranging from 160 Pa^45^ to 3900 Pa.^13^ This wide range poses a challenge in selecting appropriate parameter values for our model. Moreover, breast mechanical properties vary significantly with patient's age, menopausal status, and overall constitution. This variability underscores the need for a tailored approach to determine patient‐specific parameters. Therefore, we first focused on defining an appropriate range for the model parameters and then optimized them within this range for each case.

As a first step, we used a median‐sized breast case (M size) from a 67‐year‐old subject to represent the entire dataset, providing a balanced reference point for parameters tuning. We defined the evaluation metric as the combined distance between estimated and manually marked nipple positions on CT images, and the distance between the estimated and segmented tumor centroids. These distances were used to assess the model's accuracy in replicating the mechanical behavior of the breast.

Initially, we aimed to determine the optimal number of terms for the Prony series that would best capture the viscoelastic response of the breast. We tested up to five terms, each defined by a relaxation time ($\tau_{i}$) and modulus (*gᵢ*). We fixed the shear modulus and distributed the total relaxation parameter across terms, adjusting relaxation times to account for both short and long term tissue responses. However, initial simulations showed that a single‐term Prony series accurately replicated the observed deformation during the prone‐to‐supine transformation. Additional terms had minimal impact on model accuracy, leading us to simplify the model to improve computational efficiency without sacrificing its ability to capture key viscoelastic behavior. We therefore selected the single‐term Prony series as the final model, balancing simplicity and accuracy. While both relaxation time ($\tau_{i}$) and modulus (*gᵢ*) can be optimized, simultaneous optimization can lead to ill‐posed problems, particularly when experimental data is limited. To avoid this issue, we fixed τ to 0.01 s based on preliminary tests, which showed that this value effectively captured the dominant viscoelastic response under quasi‐static conditions.

With the number of Prony series terms determined, we proceeded to explore the optimal values for two critical parameters: the shear modulus *μ*_*B*_ (for the breast tissue) and the viscoelastic parameter *g*_*B*_. Additionally, we examined the friction coefficient *φ*, which governs interface resistance between the chest wall and the breast. We conducted simulations over a range of values for *μ*_*B*_ (80–400 Pa), *g*_*B*_ (0.1–0.9), and *φ* (0.1–0.9), aiming to minimize the combined distance metric, as previously defined.

**FIGURE 4:**
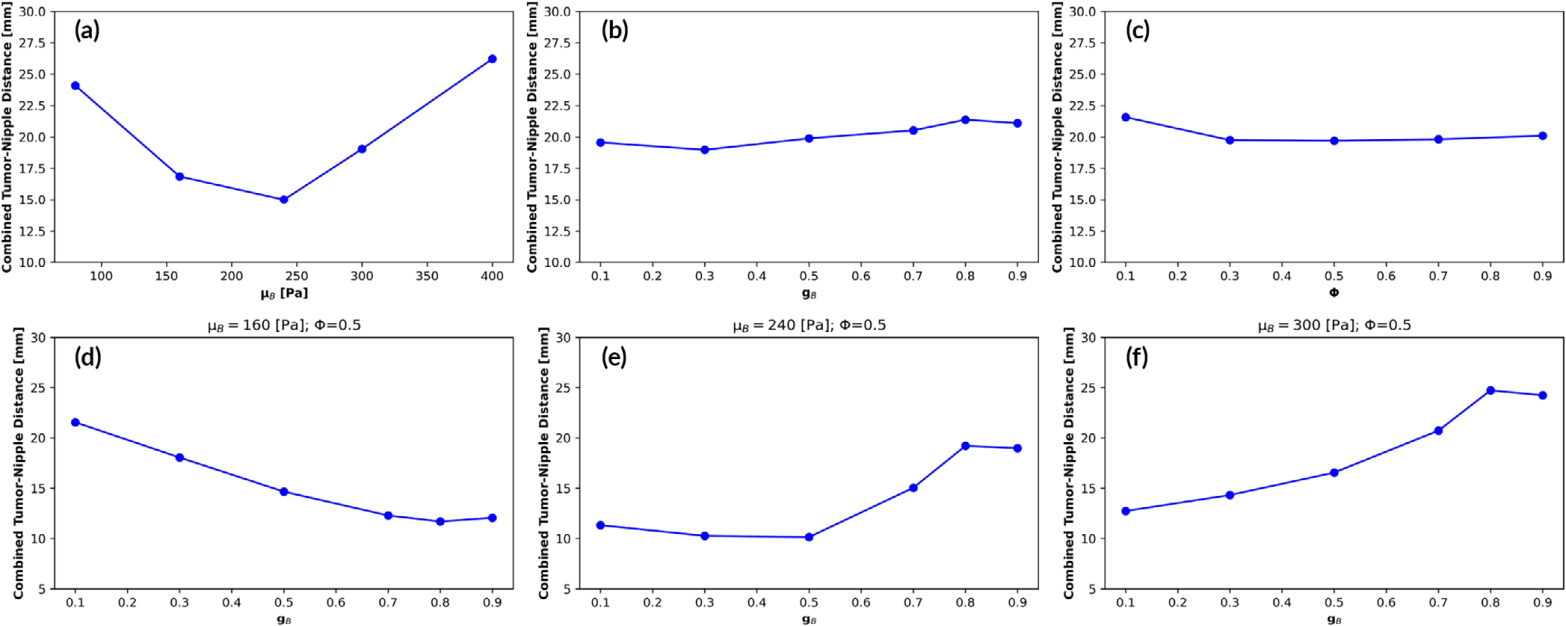
First row: (a) Combined distance of the estimated and actual nipple and tumor centroid obtained by varying *μ*_*B*_ in the range [80–400] Pa. (b) Combined distance of the estimated and actual nipple and tumor centroid obtained by varying *g*_*B*_ in the range [0.1–0.9]. (c) Combined distance of the estimated and actual nipple and tumor centroid obtained by varying *φ* in the range [0.1–0.9]. Second row: Combined distance of the estimated and actual nipple and tumor centroid obtained by varying *g*_*B*_ in the range [0.1–0.9] and fixing *φ* = 0.5 and *μ*_*B*_ = 160 Pa (d), *μ*_*B*_ = 240 Pa (e), *μ*_*B*_ = 300 Pa (f).

We gathered data from 120 simulations and determined that the optimal range for *μ*_*B*_ was between 160 and 300 Pa (Figure 4a). However, we found no significant correlation between the variations in *g*_*B*_ and *φ* and the combined distance metric (Figure 4b, c).

Based on these findings, we fixed *φ* at 0.5, a value commonly used in similar biomechanical studies.^38^

We then focused on determining the optimal value of *g*_*B*_ for the identified optimal range of *μ*_*B*_, which allowed us to narrow the parameter space. For three fixed values of *μ*_*B*_ within the optimal range, we found that the viscoelastic parameter *g*_*B*_ needed to be adjusted as follows (Figure 4d–f): *g*_*B*_ = 0.8 for 160 ≤ *μ*_*B*_ < 230, *g*_*B*_ = 0.5 for 230 ≤ *μ*_*B*_ < 300, and *g*_*B*_ = 0.1 for *μ*_*B*_ ≥ 300.

For the skin shear modulus *μ*_*S*_, we used a multiplication factor *f* applied to *μ*_*B*_, with values of *f* = [4,6,8,10,12].

Building upon the determination of the optimal material parameters range for the breast tissue, the next step was to obtain patient‐specific values through an optimization procedure. The aim was to refine the shear modulus of the breast tissue, *μ*_*B*_, to improve the model's accuracy and align its predictions with observed anatomical features.

**FIGURE 5:**
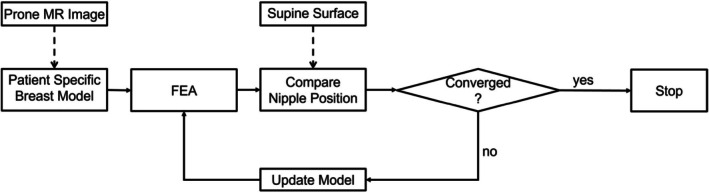
Optimization process for breast tissue material parameter *μ*_*B*_: After each finite element analysis, the predicted nipple position was compared with the actual nipple position identified in the CT image. The model was updated by refining material properties until the convergence criteria were satisfied.

The optimization metric was the distance between the predicted and actual positions of the nipple in the supine position, derived from external CT surface information. This metric guided the iterative refinement of material properties, as outlined in the workflow in Figure 5, ensuring the model converged toward the best possible fit. To solve this boundary‐constrained optimization problem, we employed a hybrid simulated annealing algorithm, *simulannealbnd*, available in MATLAB (Version 9.13, MathWorks, 2022)^53^. This algorithm adjusted the shear modulus of the breast tissue, *μ*_*B*_, within the range [160, 300] Pa, aiming to minimize the difference between the estimated nipple position, $r_{\text{nest}}$, and the reference position, $r_{n}$.

The stopping criterion for the optimization process was met when the distance between the estimated and actual nipple positions was reduced to within 5 mm. This condition is defined by the following equation:

(6) $$\text{argmin}\;\mu_{B}\;|\;r_{\text{nest}}-r_{n}\;|\;\mu_{B}\in \left[160,300\right]$$

Once the optimal shear modulus *μ*_*B*_ was identified through the optimization, we performed an exhaustive search over the predefined multiplication factor to determine the value of *f* that yielded the best nipple localization.

The model was implemented and computed on a workstation equipped with an Intel(R) Core(TM) i5‐8500 CPU @ 3.00 GHz and 16 GB of RAM. The simulations performed on this hardware exhibited an average runtime of approximately 19 min.

## 3 RESULTS

### 3.1 Estimated mechanical properties

We first present the results of the predicted shear modulus values for the breast tissue and the skin, which were obtained based on our optimization procedure within the visco‐hyperelastic model. For the breast tissue, the average shear modulus across the 15 cases was 184.2 ± 39.2 Pa, consistent with values reported by Rajagopal et al.^45^ The skin's average shear modulus was 1465.9 ± 813.4 Pa, corresponding to a multiplication factor *f* = 7.6 ± 2.9.

The viscous parameters were not optimized but were fixed based on the values of the shear modulus of the breast tissue, as determined during the parameter range analysis (described in Section 2.5).

### 3.2 Model performance in tumor localization

The performance of the model was evaluated using two metrics: tumor distance and tumor‐skin projection distance. Tumor distance refers to the Euclidean distance between tumor centroids observed in the MRI and CT images. Tumor‐skin projection distance is the distance between the skin projection of the estimated tumor centroid and the projection of the tumor's centroid from the CT image. The skin projection is defined as the closest point between the tumor's center of mass and the skin, a metric used in previous studies.^24^, ^54^ This distance plays a critical role in assessing the method's usefulness in aiding preoperative planning, as an accurate projection helps determine the most appropriate incision site.

**FIGURE 6:**
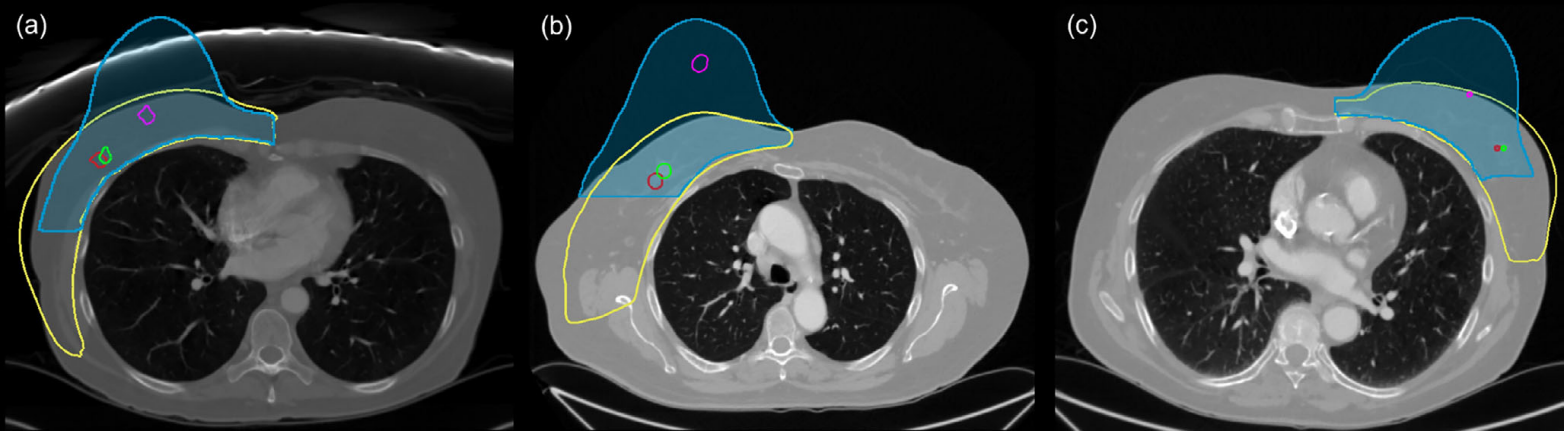
Results of the simulation with visco‐hyperelastic model and sliding contact. (a) Case 1, 56 y.o., BV = 1513.0 cm^3^. (b) Case 10, 79 y.o., BV = 2687.1 cm^3^. (c) Case 11, 63 y.o., BV = 1111.3 cm^3^. The blue region indicates the prone breast volume, and the magenta line represents the tumor in the preoperative position. The yellow line outlines the deformed mesh, while the green and red lines denote the actual and estimated locations of the tumors, respectively.

The visco‐hyperelastic model of the breast with the sliding contact condition, as defined in Section 2.4, yielded an average tumor localization error of 8.12 ± 4.15 mm. Additionally, the average distance between the skin projections of the estimated and actual tumor was 6.36 ± 4.66 mm. Figure 6 shows visual examples of the results obtained with this model.

To assess the impact of the sliding contact condition, we also evaluated the performance of the visco‐hyperelastic model with a fixed constraint at the interface with the chest wall. In this case, the average tumor localization error was 15.54 ± 5.75 mm, while the average tumor‐skin projection distance was 10.21 ± 6.21 mm.

We also tested the performance of the hyperelastic model alone, both with the sliding contact condition and the fixed constraint at the chest wall interface. For this model, the breast and skin shear moduli were optimized using the same procedure described in Section 2.5. With sliding contact, the hyperelastic model yielded a tumor localization error of 13.28 ± 7.98 mm, and a tumor‐skin projection distance of 12.84 ± 9.20 mm, indicating reduced accuracy compared to the visco‐hyperelastic model with sliding contact. Under fixed constraint at the interface with the chest wall, the hyperelastic model showed a tumor localization error of 15.34 ± 6.36 mm, and a tumor‐skin projection distance of 12.38 ± 7.07 mm, confirming that the sliding contact condition significantly improved the model's accuracy compared to the fixed constraint condition.

### 3.3 Comparison with surface‐to‐volume image‐based registration method

In this section, we present a comparative analysis of the proposed visco‐hyperelastic model and an in‐house implementation of a landmark‐driven large deformation diffeomorphic metric mapping (LDDMM) non‐rigid image registration framework.^55^ The goal of this method is to estimate the large volumetric transformation and accurately localize the tumor in the surgical position, by utilizing preoperative volumetric data from a prone MRI and supine intraoperative surface data, extracted from CT data available in the supine position.

The method begins with a rigid alignment between the preoperative volume and the intraoperative surface to establish an initial spatial correspondence. Subsequently, artificial surface fiducials are placed at specific anatomical landmarks on the breast to guide the registration process. These fiducials are used as control points to drive the landmark‐based non‐rigid registration within the LDDMM framework. The LDDMM method assumes that the landmark points at the interface with the chest wall remain fixed, and the transformation is applied to the rest of the breast tissue to estimate its deformation in the surgical position.

When this surface‐to‐volume registration method was evaluated on the same 15 cases, it achieved an average tumor localization error of 14.74 ± 4.93 mm and an average tumor‐skin projection distance of 12.49 ± 4.16 mm.

### 3.4 Summary of results

Table 2 summarizes the tumor localization performance for all evaluated models, reporting both tumor distance and tumor‐skin projection distance metrics. The visco‐hyperelastic model with sliding contact condition consistently outperformed the other approaches, achieving the lowest average tumor localization error (8.12 ± 4.15 mm) and tumor‐skin projection distance (6.36 ± 4.66 mm). In contrast, models with fixed constraints and the hyperelastic model showed higher localization errors, while the surface‐based LDDMM method yielded intermediate results.

**TABLE 2:** Comparison of tumor distance and tumor‐skin projection distance for different models.

Model	Tumor distance (mm)	Tumor‐skin projection distance (mm)
Visco‐hyperelastic/sliding	8.12 ± 4.15	6.36 ± 4.66
Visco‐hyperelastic/fixed	15.54 ± 5.75^a^	10.21 ± 6.21
Hyperelastic/sliding	13.28 ± 7.98^a^	12.84 ± 9.20
Hyperelastic/fixed	15.34 ± 6.36^a^	12.38 ± 7.07
Surface‐based method (LDDMM)	14.74 ± 4.93^a^	12.49 ± 4.16

^a^ Significant difference in comparison with visco‐hyperelastic/sliding (paired left‐tailed sign test *p* ≤ 0.05).

Additionally, we calculated the tumor localization performance for different breast volume groups using the visco‐ hyperelastic model with sliding contact. The results are summarized as follows: For the small (S) volume group, the tumor distance was 6.68 ± 3.43 mm, and the tumor‐skin projection distance was 5.50 ± 3.84 mm. In the medium (M) volume group, the tumor distance was 10.30 ± 4.70 mm, and the tumor‐skin projection distance was 8.58 ± 5.71 mm. For the large (L) volume group, the tumor distance was 7.39 ± 3.26 mm, and the tumor‐skin projection distance was 5.00 ± 3.22 mm.

Figure 6 presents visual examples of the tumor localization results using the visco‐hyperelastic model with sliding contact for different breast volumes. The figures show the deformed mesh outline, tumor locations, and the corresponding tumor projections for each case.

## 4 DISCUSSION

This study presents a novel approach to simulate the significant non‐rigid deformation of the breast from the prone to supine position using a visco‐hyperelastic material model within a FE framework. By developing patient‐specific models that adjust mechanical properties based on individual data, we ensure a more accurate representation of breast deformation. Our method, which primarily uses non‐invasive external data such as surgical surface scans, has the potential to improve preoperative localization of breast lesions.

A key innovation of this study is the use of a visco‐hyperelastic material model for optimizing patient‐specific properties. We focus on the distance between the estimated and actual nipple positions in the intraoperative scenario as the primary metric for model calibration. Although we also considered using the average distance between estimated and intraoperative surface data as a potential optimization criterion, this approach resulted in inferior performance in tumor localization and tumor‐skin projection distance. The nipple serves as a critical anatomical reference point and a robust marker of overall breast movement due to the internal structure of the breast and the convergence of the mammary ducts at this point.

The model's performance was evaluated across 15 cases, achieving an average tumor distance of 8.12 ± 4.15 mm and a tumor‐skin projection distance of 6.36 ± 4.66 mm, results that are promising for clinical applications as confirmed by clinicians involved in this study.

To assess the effectiveness of our visco‐hyperelastic model, we compared it with a hyperelastic breast model, for which the same material parameters optimization was performed. Our results show that incorporating a viscous component into the breast tissue model significantly improves tumor localization accuracy, especially regarding tumor‐skin projection distance (see Table 2).

Additionally, we evaluated the accuracy of the breast‐chest wall interface modeling by conducting simulations using both fixed and sliding contact conditions. Our findings suggest that the sliding contact condition produces a more realistic deformation and leads to enhanced tumor localization accuracy compared to the fixed constraint model (see Table 2).

We also examined the performance of the model across different breast volume groups using the visco‐hyperelastic model with sliding contact. The model exhibited the highest tumor localization error in the medium breast volume group, which was unexpected, since larger breast volumes typically result in greater deformation and higher localization errors. To explore this further, we conducted statistical analyses to assess potential contributing factors, including initial tumor displacement, estimated breast stiffness, and age. However, no statistically significant factors were identified. This suggests that the observed differences in tumor localization error between breast volume groups are not sufficiently pronounced to account for the variability, given the sample size.

We also explored the correlation between estimated breast stiffness, initial prone‐to‐supine tumor displacement, and tumor localization error across the entire population. While some moderate associations were observed, particularly with the initial tumor displacement, suggesting that larger displacements tend to result in higher localization error, this trend was not sufficient to fully explain the variability in tumor localization error.

In the current model, the breast is divided into two primary components: breast tissue (including fat, fibroglandular tissue, pectoral muscle, and Cooper's ligaments) and skin. We chose to perform an exhaustive search for the shear modulus of the skin after initial attempts to simultaneously optimize both skin and breast tissue parameters proved time‐consuming and yielded minimal improvements. This led us to adopt a more efficient approach by applying predefined multiplication factor values for skin parameters.

Our method outperforms other prone‐to‐supine registration methods that rely solely on biomechanical models and surface data, with an average landmark distance of 15.3 mm reported by Eiben et al.^25^ based on a cohort of 3 cases, and an average cutaneous distance of 20.8 mm reported by Duraes et al.^24^ in a cohort of 9 cases. Our results are comparable to methods incorporating image intensity‐based registration when a supine image (MRI or CT) is available. For example, Han et al.^15^ reported an average distance of 8.56 mm for 9 skin markers across 5 cases, while Xue et al.^21^ achieved a mean tumor distance of 8.02 mm in a cohort of 25 cases. However, Xue's study included a lower proportion of medium and large breast volume cases (25% according to our volume ranges), and smaller breasts generally exhibit less displacement from prone‐to‐supine positions, facilitating easier tumor localization with reduced errors. Our results, summarized in Section 3.4, demonstrate consistent performance across different breast sizes.

We acknowledge that our results are not directly comparable to these studies due to the lack of a public dataset, as each was evaluated on different datasets. By releasing the dataset used in this work, we aim to support the community in conducting more consistent and fair comparisons moving forward.

In this study, we identified areas of improvement in our methodology that could enhance the precision and reliability of breast deformation modeling.

One limitation of our pipeline is that the position of the nipple must be manually marked. Implementing an automatic detection algorithm for nipple localization on the intraoperative surface would be straightforward, given that the acquired surface texture includes color information. Additionally, detection algorithms for nipple localization in preoperative MRI images have already been proposed.^56^ Although we considered automating this process, the objective of this study was focused on assessing the deformation modeling rather than the automation of the full workflow including nipple detection.

Our registration process does not rely solely on nipple localization. The initial alignment also uses the automatically detected intermammary region, which serves as a stable anatomical reference (Section 2.1), to ensure accurate rigid registration between imaging space and surgical surface.

To investigate the reliability of nipple position as a stopping criterion, we performed a sensitivity analysis by simulating random perturbations of the nipple location, up to 5 mm, in five cases. The results indicated that small perturbations in nipple position, in any direction, did not significantly affect tumor localization accuracy. The mean tumor localization error remained stable across all perturbation levels, with statistical analysis confirming no significant differences. This finding suggests that using the distance to the nipple is a reliable, robust criterion for the estimation of tumor localization, and small inaccuracies or drift in its location do not compromise the overall tumor localization performance.

To evaluate the sensitivity to the stopping condition, we tested thresholds of 5, 8, and 12 mm and analyzed their impact on tumor localization errors. Statistical analysis showed no significant differences in localization accuracy across these thresholds (*p* > 0.05). These results suggest that the choice of stopping threshold does not critically influence the final accuracy of the method. Therefore, maintaining the 5 mm threshold remains reasonable, as increasing it does not significantly improve results and may affect computational efficiency and convergence stability.

Another area for improvement is the introduction of a new boundary condition addressing tissue sagging in the posterior‐lateral region. By simulating the presence of non‐deformable internal tissue in this region, we could effectively limit the extent of deformation in the postero‐lateral direction, improving the realism and accuracy of the model.

Furthermore, the accuracy of our results could potentially be enhanced by incorporating additional surface registration techniques. Methods such as affine transformations, non‐rigid iterative closest point (ICP), B‐spline‐based point registration and landmark‐driven LDDMM could refine the alignment of the external surface and contribute to a more accurate model.

Additionally, while our current analysis mainly considers gravity as the main force responsible for breast deformation, we acknowlege that the breast model was derived from a preoperative MRI, which was acquired under gravity‐loaded conditions. Future work will focus on employing methods to obtain load‐free reference states, which would enhance the model reliability.

Our modeling approach also assumes full contact between skin and breast tissue. Incorporating a sliding model between skin and breast tissue could further improve the overall accuracy of the model, especially in representing the behavior of the breast tissue during deformation and enhancing the surface data alignment.

Additionally, our modeling approach assumes a homogeneous material representation of breast tissue, which does not fully capture its inherent heterogeneity. While this simplification was necessary due to the limitations of the available imaging data, it may impact the accuracy of deformation predictions. The T2 SPAIR MRI scans used to extract the breast anatomical details did not provide sufficient contrast to reliably segment distinct tissue components such as glandular structures, Cooper's ligaments, or fascia. Consequently, extracting tissue‐specific mechanical properties was not feasible. Future work incorporating imaging modalities with improved tissue contrast (e.g., T1‐weighted MRI or specialized sequences for fibroglandular tissue differentiation) may enable the development of a more anatomically detailed and biomechanically realistic model.

A major challenge in our study is the limited availability of reliable internal anatomical markers that can be consistently identified across both breast MRI and CT images to assess model accuracy beyond tumor position. The differences in how these imaging modalities capture breast tissue, combined with the quality of the available imaging data, make it difficult to find fiducial points that can serve as consistent anatomical reference markers for target registration error. Future work will involve obtaining new imaging datasets with reliable and reproducible anatomical information, along with collaboration with expert radiologists to identify and incorporate additional anatomical markers to enhance model validation.

One further limitation is related to the variability in tumor segmentation. Tumor segmentation in our study was performed semi‐automatically on subtraction MRI images using a simple thresholding technique, which is effective given the distinct tumor boundaries. However, potential segmentation variability, whether inter‐operator or intra‐operator, may impact the accuracy of tumor localization. Future work could explore this aspect further to better assess its influence on the model's precision.

These considerations offer valuable directions for future research, as addressing these limitations could refine and advance breast deformation modeling. Incorporating these improvements could enable the method to better represent the mechanical behavior of the breast and improve the overall model accuracy and reliability.

## 5 CONCLUSION

In conclusion, the main contribution of this work lies in the development of a patient‐specific framework for modeling breast displacement from the prone‐to‐supine position using only surface information and a visco‐hyperelastic material model. The results show that the proposed approach effectively models the significant breast tissue deformation and achieves improved tumor localization accuracy compared to previous approaches. This study also highlights the feasibility of applying viscoelastic models to breast tissue, providing a strong foundation for future research in this area.

Preliminary experiments have demonstrated the feasibility of the proposed protocol for acquiring and processing surface data from optical scans in the operating room before patient preparation and draping. The only requirement is that both undraped breasts remain visible during the scan. Additionally, we confirmed that accurate nipple identification via texture mapping can be easily achieved.

Unlike previous methods relying on supine MRI or other supine images, our approach does not require modifications to the standard diagnostic imaging protocol, facilitating easier to implement in clinical settings.

Currently, the overall workflow takes approximately 20 minutes on average, primarily due to the long computation times required by our limited‐capacity workstation. Using high‐performance computing workstations could significantly reduce computation times, making this method more suitable for clinical applications and real‐time use in the operating room.

Furthermore, an accurate and reliable breast deformation model is essential to generate training data for deep learning algorithms. By simulating the same breast geometry under different loading conditions, we can generate sufficient ground truth data to train models that capture the mechanical behavior of breast tissue.^57^ These deep learning‐based deformation models could be applied in real‐time during surgical procedures, assisting surgeons with tumor localization and surgical planning.

## AUTHOR CONTRIBUTIONS

**Felicia Alfano:** Conceptualization; methodology; software; data curation; investigation; validation; formal analysis; visualization; writing – original draft; writing – review and editing. **Pedro Navas:** Conceptualization; methodology; investigation; formal analysis; writing – review and editing. **Pablo Lamata:** Formal analysis; writing – review and editing. **Karla Ferreres García:** Data curation; validation. **Juan E. Ortuño:** Formal analysis; writing – review and editing. **Oscar Bueno Zamora:** Data curation; validation. **Santiago Lizarraga:** Data curation; validation. **Andrés Santos:** Funding acquisition; project administration; writing – review and editing. **Javier Pascau:** Conceptualization; investigation; data curation; methodology; funding acquisition; project administration. **José M. Goicolea:** Conceptualization; investigation; methodology; formal analysis; writing – review and editing. **María J. Ledesma‐Carbayo:** Conceptualization; data curation; methodology; investigation; formal analysis; validation; supervision; funding acquisition; project administration; writing – original draft.

## CONFLICT OF INTEREST STATEMENT

The authors declare that they have no known competing financial interests or personal relationships that could have appeared to influence the work reported in this paper.

## Data Availability

The data underlying this study are available at (https://zenodo.org/records/17154261). The data consist of: (1) prone breast surface mesh, breast‐chest wall interface surface mesh, supine breast surface mesh, prone tumor mesh, and supine tumor mesh in stereolithography (STL) format files; (2) prone and supine nipple positions in markup JSON file format (.mrk.json).
